# A seven‐step guide to spatial, agent‐based modelling of tumour evolution

**DOI:** 10.1111/eva.13687

**Published:** 2024-05-02

**Authors:** Blair Colyer, Maciej Bak, David Basanta, Robert Noble

**Affiliations:** ^1^ Department of Mathematics City, University of London London UK; ^2^ Department of Integrated Mathematical Oncology H. Lee Moffitt Cancer Center and Research Institute Tampa Florida USA

**Keywords:** cancer, computational modelling, evolution, evolutionary medicine, population genetics

## Abstract

Spatial agent‐based models are frequently used to investigate the evolution of solid tumours subject to localized cell–cell interactions and microenvironmental heterogeneity. As spatial genomic, transcriptomic and proteomic technologies gain traction, spatial computational models are predicted to become ever more necessary for making sense of complex clinical and experimental data sets, for predicting clinical outcomes, and for optimizing treatment strategies. Here we present a non‐technical step by step guide to developing such a model from first principles. Stressing the importance of tailoring the model structure to that of the biological system, we describe methods of increasing complexity, from the basic Eden growth model up to off‐lattice simulations with diffusible factors. We examine choices that unavoidably arise in model design, such as implementation, parameterization, visualization and reproducibility. Each topic is illustrated with examples drawn from recent research studies and state of the art modelling platforms. We emphasize the benefits of simpler models that aim to match the complexity of the phenomena of interest, rather than that of the entire biological system. Our guide is aimed at both aspiring modellers and other biologists and oncologists who wish to understand the assumptions and limitations of the models on which major cancer studies now so often depend.

## 1 INTRODUCTION

Cancer initiation, progression and treatment responses are Darwinian evolutionary processes (Casás‐Selves & DeGregori, 2011; Merlo et al., 2006) that can be investigated using a wide range of mathematical and computational methods. Examples include evolutionary game theory (Basanta et al., 2011; Yang et al., 2016), branching processes (Danesh et al., 2012; Durrett et al., 2010), and Moran processes (Durrett et al., 2016; West et al., 2016). Yet while many tools have yielded important insights into cancer evolution, the study of spatial aspects—especially important in carcinomas, constituting the majority of humans cancers—often necessitates a spatially explicit approach, such as a spatial agent‐based model.

An agent‐based (or individual‐based) model is a computational model of a system made up of autonomous, interacting “agents”. Spatial agent‐based models (SABMs) have long been used to study the evolution of spatially structured communities because they can reveal how the processes of selection, drift, and gene flow depend on localized interactions among agents (typically individual organisms) or between agents and their spatially varying environment. As new technologies generate better spatial tumour data, SABMs are proving ever more useful in oncology. Typical applications include understanding tumour development, inferring the effects of driver mutations, and predicting treatment outcomes. For example in recent studies, Aif et al. (2022) used an SABM to investigate the evolutionary rescue of drug‐resistant tumour subclones; Saha et al. (2023) used an SABM to investigate adaptive cancer therapy; and Bull and Byrne (2023) used an SABM to simulate interactions between macrophages and tumour cells.

To support this burgeoning research field, here we present a seven‐step guide to designing and implementing spatial agent‐based models in which the agents are locally interacting tumour cells or cell subpopulations. Starting from the simplest cellular automata, we discuss options for adding greater complexity and biological realism, such as multi‐level spatial structure and environmental heterogeneity. Based on our extensive experience of developing and using SABMs (Bacevic et al., 2017; Bak et al., 2023; Noble et al., 2020, 2022), we cover practical issues such as event scheduling, visualization, and how to use SABMs to infer parameter values from experimental or clinical data. Each topic is illustrated with examples from our own demon‐warlock modelling framework (Bak et al., 2023; Noble et al., 2022), other state of the art modelling platforms, and studies that have used SABMs in cancer research. Whereas our focus is on tumour evolution, much of our advice applies equally to similar modelling methods used to study bacterial colonies, invasive species and organismal development. The guide is designed to be accessible for biologists and clinicians without specialist mathematical knowledge.

## 2 SPATIAL STRUCTURE

Spatial structure determines the evolutionary balance between selection and drift, the nature of gene flow between subpopulations, and the strength of ecological interactions. When a model fails to accurately represent the spatial structure of a biological system, the model's predictions and inferences for that system may be highly unreliable (Noble et al., 2022; Strobl et al., 2022). It follows that the parameters of spatial structure—such as the size of locally interacting cell communities and the manner of cell dispersal—should be accorded the same importance as evolutionary parameters in model design. Notwithstanding the trade‐off between model simplicity and realism, spatial structure parameters should, as far as possible, be derived or inferred from empirical data.

### 2.1 Stochastic cellular automata

**FIGURE 1:**
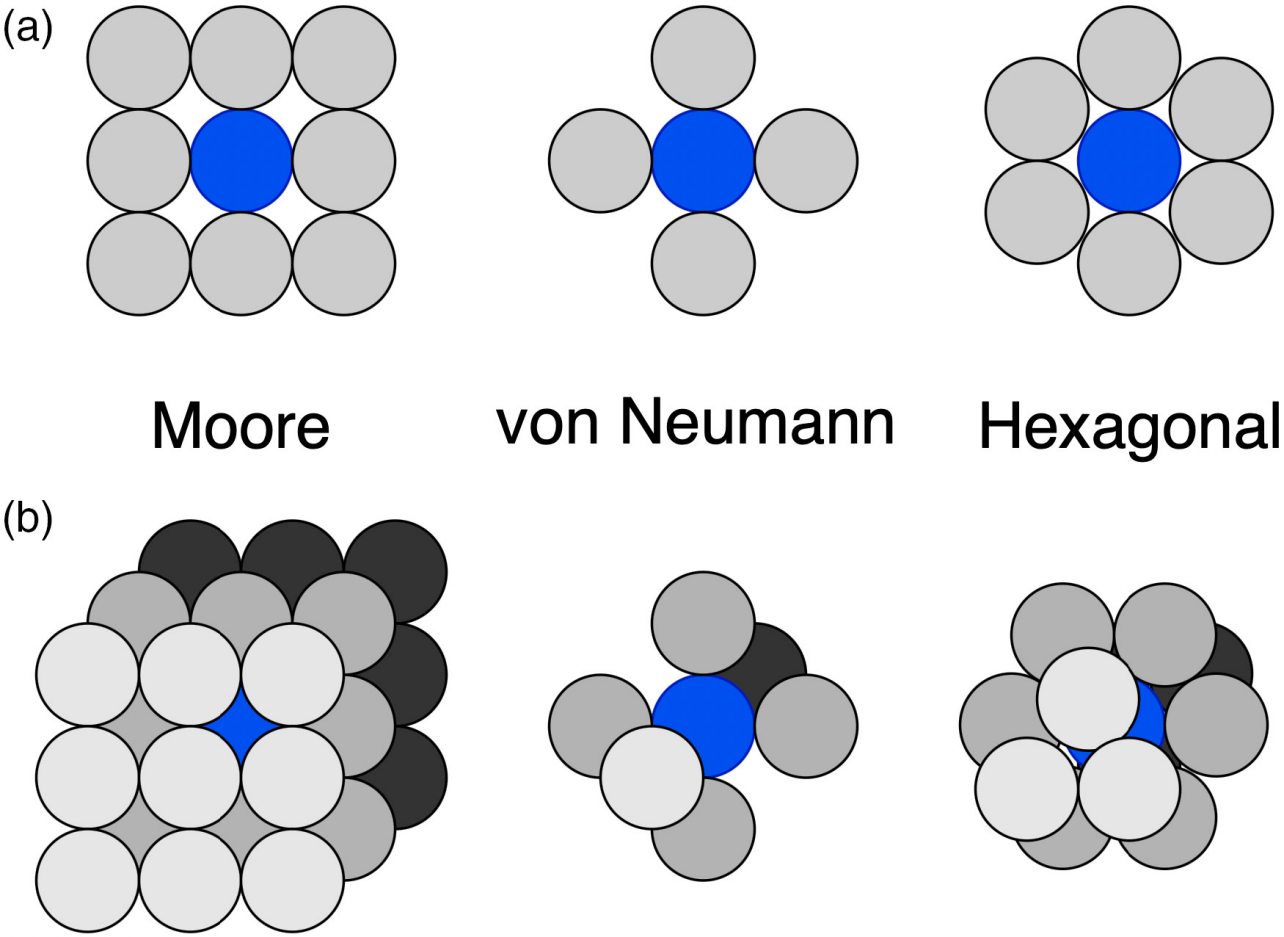
Some common neighbourhoods that govern the update rules for cellular automata and other agent‐based models in two dimensions (a) and three dimensions (b). A focal agent (cell) is shown in blue and its neighbourhood sites in grey.

Many of the simplest spatial agent‐based models are cellular automata. A cellular automaton is a model that plays out on a grid of sites in one or more dimensions. Each site is associated with one of a set of at least two possible states. Each site also belongs to a subset of sites called a neighbourhood, of which some examples are shown in Figure 1. For example, the von Neumann neighbourhood in two dimensions contains the nearest sites in the cardinal directions (up, down, left and right). A cellular automaton sequentially updates itself according to a set of rules. The update rules for a given site depend on its own current state and the states of the sites in its neighbourhood.

Whereas the update rules of many cellular automata are deterministic (Schiff, 2011), probabilistic rules are more appropriate for modelling stochastic processes such as biological evolution. A stochastic cellular automaton is equivalent to a collection of locally interacting Markov chains, which means that each event is chosen according to probabilities that depend only on the current model state, not any of its previous states.

In biological terms, each state corresponds to a type of cancer cell or some other entity (such an immune cell or part of the extracellular matrix). Generally we will assume that the focal agents in our models are cancer cells and we will use the terms “agent” and “cell” interchangeably where appropriate. A cellular automaton permits a cell's event probabilities (for example, its division, death and dispersal rates) to depend on the number of neighbouring cells. This allows us to account for crowding or Allee effects, such that birth, death or dispersal rates depend on the local or global population size. Event rates can also vary according to the types of the neighbouring cells, for example to simulate cell competition or immune predation.

Models of asynchronous processes, such as cell division in a tumour, typically use asynchronous updating, meaning that only one or a small number of sites are modified per update (Louis & Nardi, 2018). In addition to being more realistic, asynchronous updating is often necessary to prevent conflicts. For instance, if two cells are attempting to divide but only one space is available for the two potential daughter cells then one must take priority.

### 2.2 The Eden growth model

Among the simplest stochastic cellular automata is the Eden growth model. This model is typically implemented on a two‐ or three‐dimensional regular square grid with only two possible states: unoccupied ($S_{0}$) and occupied ($S_{1}$). With each iteration, the update rule causes a site in the neighbourhood of an $S_{1}$ site to switch from $S_{0}$ to $S_{1}$. In this way new $S_{1}$ sites (cells) are added to the surface of a cluster. The Eden growth model on an n‐dimensional grid self‐organizes to resemble an n‐dimensional ball with a non‐trivial surface. The growth curve of the $S_{1}$ population approaches a polynomial of degree n (Eden, 1961).

The three most popular options for the Eden growth model update rule can be labelled alphabetically:

1. **A**vailable site‐focussed: Choose at random an $S_{0}$ site in the neighbourhood of an $S_{1}$ site, and switch it from $S_{0}$ to $S_{1}$.
2. **B**ond‐focussed: Choose at random an $S_{1}$ site with a probability proportional to the number of $S_{0}$ sites in its neighbourhood, and then randomly choose an $S_{0}$ neighbour and switch it to $S_{1}$.
3. **C**ell‐focussed: Choose at random an $S_{1}$ site with at least one $S_{0}$ site in its neighbourhood, and then randomly choose an $S_{0}$ neighbour and switch it to $S_{1}$.

Although these update rules result in similar large‐scale patterns, they generate cluster surfaces with different microscopic properties. Indentations in the model surface are more likely to be filled, and spikes are less likely to form, under option C than under option B, and under option B than under option A. Hence option C generates the smoothest surface and option A the roughest (Jullien & Botet, 1985).

Variants of the Eden growth model have been used to investigate the evolution of paediatric glioma (Tari et al., 2022), colon cancer (Hamis, Yates, et al., 2021), hepatocellular carcinoma (Lewinsohn et al., 2023; Waclaw et al., 2015), clear cell renal cell carcinoma (Xiao et al., 2022) and non‐small cell lung cancer (Jagiella et al., 2016). Many studies use a variant that includes stochastic cell death. By opening up spaces for cell division, cell deaths increase clonal mixing within the tumour and facilitate selection (Waclaw et al., 2015).

### 2.3 Other grid‐based stochastic cellular automata

Other stochastic cellular automata can be more appropriate than the Eden growth model for modelling systems in which state changes are not confined to the surface. Spatial branching processes are similar to Eden growth models except that if a dividing cell has no space to divide then it can create space by budging other cells. An intermediate model can be created by stipulating that only nearby cells can be budged, so as to simulate physical constraints on cell division. Chkhaidze et al. (2019) recently used such a model to investigate how spatially constrained tumour growth alters signatures of clonal selection and genetic drift in cancer genomic data. Good practice is to implement budging along an approximately straight line between the dividing cell and the nearest empty site. If budging is instead restricted to the cardinal directions or the cardinal and intercardinal directions then the simulated tumour will self‐organize into an approximate square or octahedron, rather than a more biologically plausible disc or ball.

Another option is to allow dividing cells to replace, rather than displace, their neighbours. In the voter model, the update rule is such that, with a certain probability, a randomly selected site copies the state of a neighbouring site. Biasses can be introduced by setting unequal copying probabilities, corresponding to differences in cell fitness. Simple (linear) voter models satisfy a convenient property called coalescing duality, which means that their typical behaviour can be explained through mathematical analysis (Durrett, 2007). In a pioneering 1972 study, Williams and Bjerknes (1972) used a biased voter model to simulate the spread of skin cancer through the basal epithelial layer.

**FIGURE 2:**
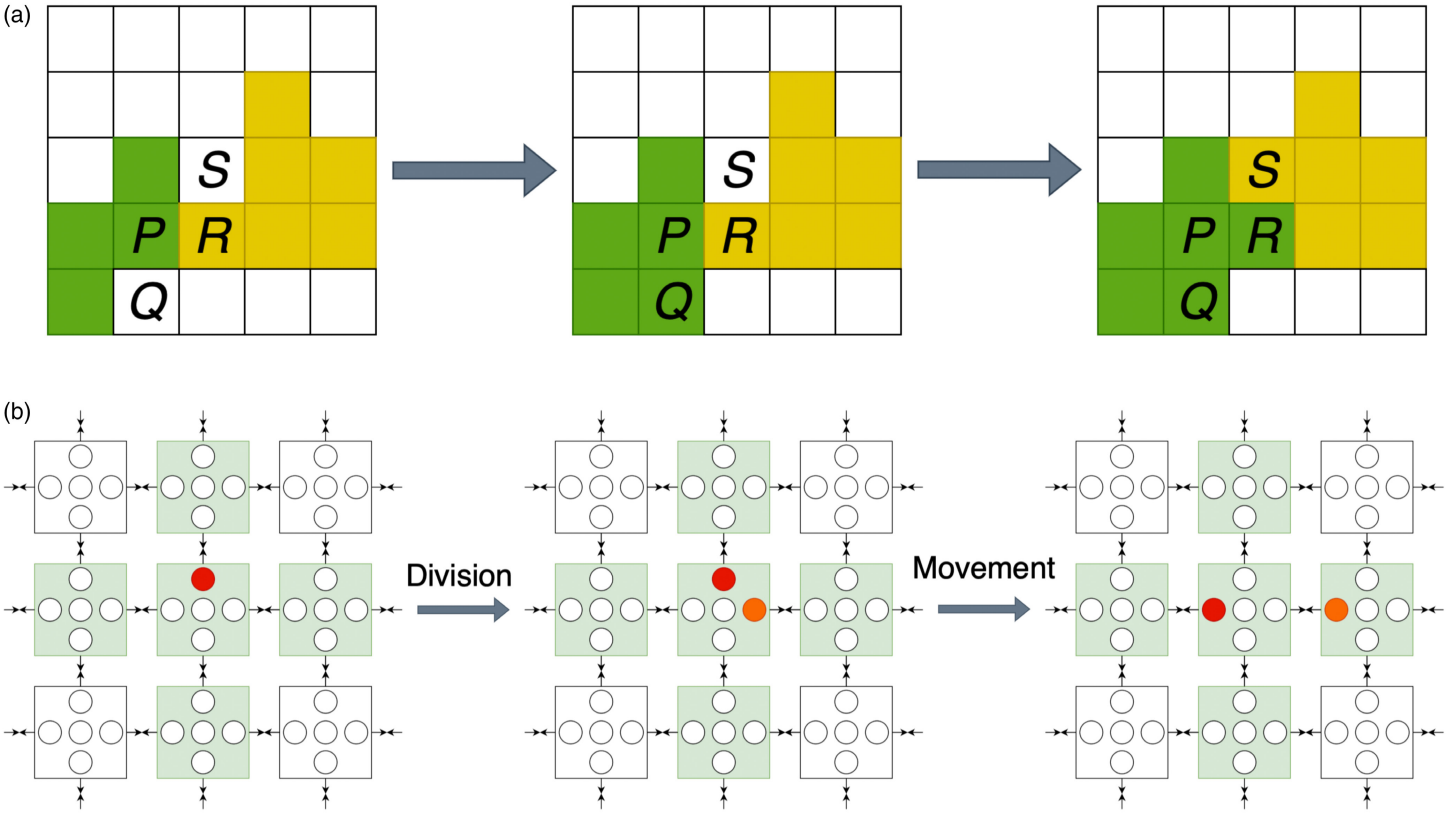
(a) An illustration of the CPM. Here, two cells, shaded in green and yellow, each occupy multiple sites on a grid. In the leftmost panel, we see the model's initial configuration; in the middle panel, the state of site P is copied onto site Q, and the green cell grows; in the rightmost panel, the state of site P is copied onto site R, which is initially occupied by the yellow cell, thus deforming the yellow cell and budging it into site S. (b) An illustration of the LGCA. Grid sites shaded in green represent those which may influence the focal cell node, shaded in red. The leftmost panel represents the initial configuration; the middle panel shows a cell dividing into free space on its grid site, with the new cell shaded in orange; the rightmost panel demonstrates how cells might move within a grid site or between grid sites: the red cell has changed direction, and the orange cell has moved from one site to another.

The cellular Potts model (CPM), also known as the Glazier‐Graner‐Hogeweg model (Graner & Glazier, 1992; Savill & Hogeweg, 1997), more explicitly simulates physical interactions among cells and between cells and their microenvironment. The model takes place on a lattice and each cell is represented by multiple lattice sites (as opposed to only one lattice site, as in previously discussed models), corresponding to the cell's volume (Figure 2a). Cells are deformable and can adhere to one another or to surrounding empty sites (which might represent extracellular matrix or growth medium). Hamiltonian mechanics describe the overall energy of the system depending on adhesion forces and resistance to changes in cell volume. A random lattice site is chosen at each time step and its state is copied to a random neighbouring site. If the new configuration has lower energy than the previous configuration then the change is always accepted; otherwise, the probability of accepting the change depends on the Boltzmann temperature. The CPM has been used in numerous cancer studies, such as for simulating tumour growth, invasion and evolution (Szabó & Merks, 2013), or for investigating how cell compressibility, motility and contact inhibition shape tumour cell clusters (Li & Lowengrub, 2014). The CompuCell3D modelling environment compucell provides an efficient, flexible CPM implementation.

The biological lattice gas cellular automaton (Deutsch & Dormann, 2005) excels instead at modelling cellular movement, and especially collective migration, in a simple, computationally efficient and physically correct fashion. The model must play out on a square or hexagonal lattice in 2 dimensions, or a cubic, dodecahedral or icosahedral lattice in 3 dimensions. States incorporate cell velocities. For instance, consider a 2‐dimensional square lattice in which each site contains 5 nodes: one for each directional velocity and a resting node at the centre (Figure 2b). A cell occupying any one of these nodes can divide into other nodes on the same site. A cell can also reorient itself by moving between nodes on the same site and can move between sites according to its velocity, provided there is space to do so. This model has been used, for example, to give insights into breast cancer invasion plasticity (Deutsch et al., 2021).

### 2.4 Multi‐level spatial structures

An important limitation of all the aforementioned cellular automata is that their uniform spatial structures are inconsistent with the biology of many tumour types. Various common cancers have glandular structures and grow via individual cells or small cell clusters invading neighbouring tissue (Lugli et al., 2021; Pandya et al., 2017). Colorectal adenomas are also glandular but grow through gland fission (Preston et al., 2003).

Inspired by classical population genetics models (Moran, 1958), a simple, conventional way to account for multi‐level spatial structure in tumours is to assign cells to local subpopulations, called demes, located on a regular grid. Thus each grid site is allowed to contain not only one but dozens, hundreds, or thousands of cells. The subpopulation size per deme is prevented from exceeding a certain threshold—known as the deme's carrying capacity—by decreasing cell division rates or increasing death rates as the subpopulation size grows.

Deme‐based models allow for more complicated modes of cell dispersal. As in the voter model, cells can be assigned some probability of invading neighbouring demes, either individually or in clusters. The dispersal probability can also be made to depend on the population of the deme being invaded, so that cells disperse more easily in less densely populated regions near the tumour periphery. Alternatively, each occupied deme can be assigned a probability of undergoing fission, resulting in some of its cells being moved to an unoccupied neighbouring deme. Depending on the degree of budging allowed, the deme‐level dynamics of the fission model can resemble an Eden growth model (no budging of demes) or a spatial branching process (unlimited budging). Deme‐based models additionally allow for the explicit simulation of tissue invasion, such that a tumour can grow only via its cells invading demes that are initially filled with normal cells (Noble et al., 2022).

### 2.5 Aggregating agents

If the within‐deme subpopulations can be assumed to be well‐mixed then cells that belong to the same deme and have the same phenotype and genotype can be modelled collectively, rather than as individual agents. This model design not only improves computational efficiency but can also facilitate mathematical analysis. For example, when cells disperse by invading neighbouring demes, the model can be designed so that the dynamics are approximately equivalent to the well understood spatial Moran process (Noble et al., 2022). Cells can be randomly selected within a deme by sampling from a hypergeometric distribution.

Even greater efficiency can be realized by not modelling inter‐deme dynamics at all, and simply making the demes themselves the model agents (Siegmund et al., 2009; Sottoriva et al., 2015). Although such coarse‐graining enables the simulation of much larger tumours, it comes at the cost of reduced precision. Care should be taken in translating between mutation rates per cell and effective mutation rates per deme.

### 2.6 Off‐lattice models

Instead of confining agents to a regular grid, we might instead locate them in continuous space. This structure is potentially more realistic but also entails more parameters, more decisions to be made, and typically higher computational costs (Beerenwinkel et al., 2015). To prevent multiple cells occupying the same space and to maintain tumour integrity, we now must model the movement of cells in response to physical forces such as cellular adhesion and repulsion (Franz et al., 2014). We may also choose to model directed movement under the influence of diffusible factors (hapotaxis).

There are several practical ways to prevent cells overlapping in an off‐lattice model, depending on how the agents are implemented. Suppose we have spherical cells, each with fixed radius r. We can then specify that when, as a result of cell division or movement, the distance between two cells' centres is less than 2r, both cells will simply be pushed in opposite directions. Alternatively, to account for cell deformation, we might implement repulsion only when the distance between cell centres falls below some threshold value smaller than 2r (Macklin et al., 2012). Some modelling platforms achieve greater realism and tractability by implementing adhesion and repulsion forces using functions rooted in physics, which are beyond the scope of this guide (see documentation cited in the Appendix S1).

## 3 MUTATION

Having chosen an appropriate spatial structure, we next will decide which cell phenotypes and genotypes to include in our state space, and how to model mutations between these states. As ever, the goal is to balance model simplicity, realism and computational demands.

### 3.1 Defining phenotypes

A good part of the difficulty in designing a useful model stems from the fact that much of the experimental data gathered by cancer biologists focusses on genetic mutations while the rules that govern the behaviour of the agents in an SABM assume an understanding of the key cancer phenotypes. The most basic actions a tumour cell might perform at any given time step are apoptosis/death, proliferation and motility. These are often considered as simple probabilistic events and often modelled in a exclusionary manner, so that if a cell is moving then it is neither proliferating nor dying. The required probabilities can either be taken directly from experimental data (which is often hard to measure in vivo and unrealistic in vitro) or calibrated with in vivo pre‐clinical models.

Using hard‐coded rules to model the phenotype of a tumour cell, while relatively simple, does not capture the flexibility shown by biological cells in the mapping between genotype and phenotype. Gerlee and Anderson (2009) have instead proposed capturing some of the complexity of this mapping by embedding neural networks inside each agent, so that the phenotype emerges in a non‐linear way as a result of the agent's state and the different microenvironmental inputs to which the agent is receptive.

### 3.2 Trait evolution versus population (epi)genetic models

Once phenotypes have been defined, the next step is to determine how these phenotypes will change as a result of mutations. One option is to model mutations as phenotypic switches. Many studies consider models with only two possible tumour cell states—mutated and unmutated—which differ in fitness (Sottoriva et al., 2015), degree of drug resistance (Gallaher et al., 2018), or some other trait. Grow‐or‐go models assume that cells can reversibly switch between predominantly migratory and predominantly proliferative phenotypes (Hoek et al., 2008). Other models examine the evolution of continuous traits, such as levels of glycolysis and acid production (Robertson‐Tessi et al., 2015).

If we are more interested in clonal dynamics then we can explicitly track changes to the (epi)genome. These mutations are conventionally assigned to three groups according to how they affect cell fitness: driver mutations (which increase cell fitness), passenger mutations (no effect), and deleterious mutations (negative effect). For simplicity, most studies assume an infinite sites model (Kimura, 1969), such that no two mutations can occur at the same site. Finite sites models must be parameterized based on observed mutation frequencies (Schenck et al., 2022).

### 3.3 Example: The Eden growth model with mutation

**FIGURE 3:**
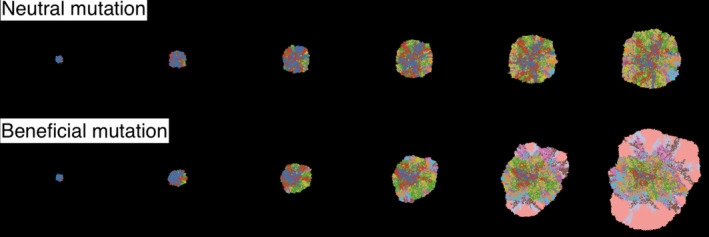
The result of running an Eden growth model with nearly neutral mutations (top) and beneficial mutations (bottom). Model produced in HAL using some in‐built examples as a skeleton for the code (West, 2018).

We can convert an Eden model into an evolutionary model by implementing mutation. The grid and neighbourhood are defined as before but now we have multiple cell states $S_{1},S_{2},S_{3},\ldots$ and mutation rates between each pair of distinct cell states. A simple option, assuming infinite sites, is to set all mutation rates to be zero except in the case of $S_{i}$ to $S_{i}+1$ for all i≥0, so that every $S_{i}$ cell has exactly i mutations. Let us assume that all these mutations are drivers and their effects combine multiplicatively, such that each mutation increases the division rate by a factor of 1+s, with s≥0. Assume also that mutations occur only at the time of cell division, and the number of new mutations per daughter cell is Poisson distributed. We then arrive at a reasonable toy model of spatial tumour evolution that can be implemented in not much more than 100 lines of code, as we illustrate with an R script (Noble, 2018). Figure 3 shows results of implementing a similar model in the HAL platform (West, 2018).

### 3.4 Distributions of fitness effects

Modelling the evolution of a quantitative trait, such as cell division or death rate, leads to further design decisions. As in our toy model, it can be wiser to draw mutation fitness effects from a probability distribution instead of setting them all equal. To see why, consider a model of an expanding tumour that, in the absence of mutation, has radius growth rate $c_{0}$, and in which the spread of mutants is not confined to the periphery (for example, a biased voter model). When a new mutant arises within the wildtype population, its long‐term fate, in the absence of further mutation, will be sensitive to its radius growth rate, $c_{1}$. If $c_{1}<c_{0}$ then the mutant will remain forever rare; if $c_{1}>c_{0}$ then the mutant is bound to take over the entire tumour; if $c_{1}=c_{0}$ then the mutant will become relatively more abundant over time without ever fully replacing the wildtype. Randomising the fitness effect randomizes $c_{1}$ and so randomizes mutant fates. Our demon‐warlock framework draws each selection coefficient (relative increase in cell division rate) from an exponential distribution.

Strictly multiplicative fitness is best avoided in all but the smallest‐scale models as it can lead to unrealistically high fitness values. This is especially problematic if mutation is implemented at the point of cell division, which creates a feedback loop in which lineage fitness grows at an ever increasing rate. A simple solution implemented in our demon‐warlock framework is diminishing returns epistasis. When the selection coefficient of a driver mutation is s, instead of multiplying the division rate by 1+s, we instead multiply by $1+s\left(1-b/b_{\max}\right)$, where b is the previous division rate and $b_{\max}$ is an upper bound.

## 4 EVENT SCHEDULING

The next step is to consider how to implement cell events algorithmically. Event scheduling can be the most important factor in determining computational efficiency, especially in simpler grid‐based models. The optimal choice strikes a balance between efficiency, simplicity and biological realism.

### 4.1 Gillespie's algorithm

The Gillespie Stochastic Simulation Algorithm (Gillespie, 1976) is an especially simple and popular solution to event scheduling. Event rates are assumed to depend only on the current state of the model and the time between events is exponentially distributed (as in a Poisson process), such that two events cannot occur simultaneously. The steps of the algorithm are as follows:

1. Initialize the system.
2. Set event rates (birth rates, death rates, dispersal rates, etc.).
3. Randomly determine the next event such that $\mathbb{P}\left(\text{event}=E\right)=$ $\text{rate}\left(E\right)/\Sigma \left(\text{rates}\right)$.
4. Implement the chosen event.
5. Advance the timer by $\mathrm{\delta t}∼\mathrm{Exp}\left(1/\Sigma \left(\text{rates}\right)\right)$.
6. Repeat from step 2 until a stop condition is reached.

This algorithm is more efficient than the event timer approach (see below) and is very easy to implement. In statistical terms, the simulated sequence of events corresponds to a trajectory of a set of stochastic differential equations, called the master equations. This means we have a good mathematical understanding of how the algorithm behaves.

Our toy Eden growth model (Noble, 2018) provides an example implementation of Gillespie's algorithm. This model further improves computational efficiency by keeping track of the cells that have space to divide, so that the next dividing cell can be chosen from among this subset (which in n dimensions scales with the radius to the power of n−1) rather than from the entire cell population (which scales with the radius to the power of n). The drawback is that cells without space to divide never undergo mutation, which may be an unjustifiable assumption in a serious research model.

Modifications of Gillespie's algorithm, such as tau leaping (Gillespie, 2001), are even faster but less accurate. Tau leaping allows multiple events to occur simultaneously, which may be problematic in a spatial model if the events affect multiple sites in close proximity (for example, if two cells are chosen to divide into the same empty site). Moreover, tau leaping improves performance only when the system is dominated by a small number of large, homogeneous subpopulations, which is typically not the case in SABMs.

### 4.2 Gillespie's algorithm with phase‐type distributions

A shortcoming of the Gillespie algorithm is that some events, such as cell division, are not true Poisson processes with exponentially distributed waiting times. In effect, the Gillespie algorithm permits arbitrarily short cell cycles. Some cells may divide several times while, in the same period, others with identical division rates fail to divide at all.

One way to achieve more realistic cell cycle periods without sacrificing very much computational efficiency is to use a phase‐type probability distribution. Whereas an exponential distribution models the time until the next event in a Poisson process, a phase‐type distribution models the time taken for an entire sequence of events, which may occur at different rates.

In practical terms, this entails executing the Gillespie algorithm as above, except that when a cell is selected for division, it doesn't necessarily divide immediately, but instead changes its position in the cell cycle. Given a target probability distribution for cell cycle periods, we can use an algorithm to choose transition rates such that the resulting phase‐type distribution has the same mean, variance and skew as the target (Osogami & Harchol‐Balter, 2006). For example, suppose that all cells begin in division state 0. When a cell is selected (according to a state‐dependent probability), its state is updated from 0 to 1, 1 to 2, or 2 to 3. When a state 3 cell is selected it divides and both progeny are reset to state 0 (Belluccini et al., 2022). The method's greater realism comes at the cost of additional memory demands and longer execution time, compared to the basic Gillespie algorithm.

### 4.3 Random sampling with binary trees

**FIGURE 4:**
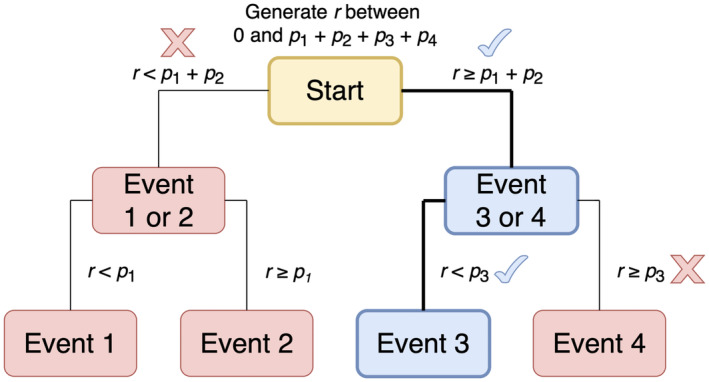
An example of using a binary tree to select an event (Event 3) from four options. Selected nodes are shown in blue.

When we have more than a handful of events to choose from it will be much more efficient to implement event selection using a binary tree. Suppose, for example, that we have four possible events with rates $p_{1},p_{2},p_{3}$ and $p_{4}$, where $p_{1}\leq p_{2}\leq p_{3}\leq p_{4}$. If we store the rate sums $p_{1}+p_{2}$, $p_{3}+p_{4}$, and $p_{1}+p_{2}+p_{3}+p_{4}$ then we can choose an event as follows. First we generate a random number r from a uniform distribution between 0 and $p_{1}+p_{2}+p_{3}+p_{4}$, and we examine whether $r<p_{1}+p_{2}$. Supposing r is greater than $p_{1}+p_{2}$, we then test whether it is less than $p_{3}$. If so then we choose event 3; otherwise event 4. Effectively, we have traversed a binary tree, beginning at the root node associated with the sum of all event rates, and ending at a terminal node associated with a single event (Figure 4).

The binary tree method is efficient because both the number of steps needed to choose an event, and the number of nodes that need updating following a change in an event rate, grow only with the logarithm of the number of possible events. For example, we need only 20 steps to choose between one million possible events. As long as the cell population keeps growing, there is little benefit to pruning nodes and it is easy to ensure that the tree remains balanced. The rate sums together take up only as much computer memory as the individual rates. The main costs are in terms of code development time and code complexity. Binary trees require careful implementation and error checking to ensure that existing nodes are updated and, when required, new nodes are added after each model event. Our demon model implements binary trees and periodically recalculates event rate sums to prevent excessive accumulation of rounding errors.

### 4.4 Cell cycle timers

A less efficient alternative to using phase‐type distributions is to draw inter‐division times directly from a chosen probability distribution. This approach enables more precise tracking and adjustment of individual cell cycles. An algorithm used in recent studies (Gallaher et al., 2018; Robertson‐Tessi et al., 2015) is as follows:

1. Initially assign every cell i a countdown timer set to time $t_{i}$ drawn from some probability distribution (dependent on the cell's phenotype).
2. Subtract δt from every countdown timer, where $\mathrm{\delta t}\ll t_{i}$ for all i.
3. For all cells i, in random order:
  - Implement cell death and dispersal events for i;
  - If i is alive, has space to divide, and $t_{i}\leq 0$, then i divides;
  - Assign each new cell a countdown timer, set to some random time dependent on the new cell's phenotype.
4. Repeat from step two until a stop condition is reached.

How much this approach reduces computational efficiency will depend on other aspects of the model. It is likely to be much slower than a well implemented Gillespie algorithm when applied to a simple grid‐based model, due to the additional burdens of updating every cell (Step 2) and shuffling all the cells (Step 3) at each small time step. In an off‐lattice model, where cells move much more frequently than they divide, and where a shuffling algorithm may already be required to randomize the order in which cell positions are updated, the cost of updating cell division state at the same time as position may be negligible.

## 5 MICROENVIRONMENT

Whereas many SABM studies focus on the effects of spatial structure and cell–cell interactions, real tumours evolve in a complex microenvironment that varies over space and time. This tumour microenvironment, comprising both molecular elements, such as cytokines, and other (non‐cancer) cells, constitutes the cancer ecosystem (Anderson & Simon, 2020)—a key element of the selection process driving somatic evolution. Given a good rationale and sufficient parameterization data, we may choose to extend our model by explicitly simulating microenvironmental factors in the form of agents (in the case of immune cells or stromal cells) or diffusible factors (such as oxygen and drugs). Permitting cancer cells to modify their selective environment creates potential for emergent complexity and niche construction (Chaplain & Anderson, 1996; Qi et al., 1993).

### 5.1 Hybrid cellular automata

Hybrid cellular automata (or HCA) have been used to model interactions between tumour cells and diffusible factors for more than 20 years. As described in a pioneering 2001 paper by Patel et al. (2001), these models consist of two interdependent components: stochastic cell events, and deterministic reaction–diffusion partial differential equations. The latter component dictates how chemicals or other factors work their way through the system as they are consumed and processed by cells. Local concentrations of diffusible factors contribute to the cell update rules.

Typically we assume that diffusible factor concentrations rapidly re‐equilibrate following changes in the configuration of cells. We can then numerically solve the equations to find the equilibrium concentrations either after every cell event or, trading some accuracy for greater efficiency, after a relatively small number of cell events have occurred. Suitable procedures for solving partial differential equations as initial value problems can readily be found in textbooks and software libraries. These range from simple but inefficient algorithms based on the classical Gauss–Seidel method, which require only a few dozen lines of code (Bacevic et al., 2017; Patel et al., 2001; Press, 2007), to the highly sophisticated BioFVM solver (Ghaffarizadeh et al., 2016), which is specifically optimized for hybrid SABMs. Several SABM platforms include their own methods for solving reaction–diffusion equations in two or three dimensions (see Appendix S1).

### 5.2 Types of diffusible factor

To add biological realism, we might make cell division and death rates in our model depend on the local oxygen and glucose concentrations as these factors diffuse through the tumour from the surrounding medium (in very small tumours and tumour spheroids) or from point sources representing blood vessels (in larger, vascularized tumours). We might also modify dispersal rates so that cells follow oxygen or glucose gradients. Potential adverse factors include acid produced through tumour cell metabolism, and drugs that diffuse from blood vessels. Hybrid cellular automata are especially suitable when the supply of an influential factor is highly variable over space or time, such as in the case of intermittent drug treatment (Bravo et al., 2020).

## 6 PARAMETERIZATION AND INFERENCE

Although theoretical models can be valuable for generating hypotheses and providing proof of concept, if we want to apply an SABM to studying a particular biological system then we must ensure that its influential parameter values are set appropriately. Parameterization should ideally be based on clinical or experimental data specific to the biological system of interest; otherwise values can be estimated from studies of similar systems or theoretical considerations (for instance, diffusion coefficients approximately correlate with molecular weight). Influential parameters might pertain to the effects of mutations, drugs, oxygen and glucose; rates of chemical supply, diffusion, consumption and decay; cell dispersal modes and rates; baseline cell death rates, crowding effects and the size of interacting cell communities. Since calibrating SABMs is often computationally demanding, high‐performance computation may be required to generate the necessary resources to calibrate them properly.

### 6.1 Example: Hybrid cellular automaton for simulating a tumour spheroid

Bacevic and Noble et al (Bacevic et al., 2017) parameterized a HCA to mimic tumour spheroid evolution under drug treatment. In spheroids the limiting factor for cell survival and proliferation is oxygen. Other diffusible factors such as glucose were therefore omitted to simplify the model without compromising its usefulness. The oxygen concentration in the medium and oxygen diffusion rates were drawn from previous studies (Casciari et al., 1992a; Grimes, Kelly, et al., 2014; Kim et al., 2007), as were the mathematical relationships between oxygen consumption rate, cell proliferation rate and local oxygen concentration (Casciari et al., 1992b; Grimes, Fletcher, et al., 2014). The different maximum proliferation rates of drug‐sensitive and resistant cells, reflecting a fitness cost of resistance, were determined from new monolayer growth assays. Cells with insufficient oxygen supply were assumed to die.

Since oxygen effects alone fail to account for the extent of quiescence observed in tumour spheroids, Bacevic and Noble et al implemented crowding effects by permitting cell budging only within a specified radius. New monolayer growth assays revealed that the relationships between cell proliferation rate, death rate and drug dose could be well approximated with piecewise linear functions. The drug's impact on proliferation was further assumed to multiply the oxygen effect, consistent with prior observations (Casciari et al., 1992b). Drug consumption was also modelled using Michaelis–Menten kinetics, with a diffusion rate chosen according to the drug's molecular weight and an appropriately low consumption rate. Thus parameterized, the SABM accurately predicted the outcomes of new tumour spheroid experiments (Bacevic et al., 2017).

### 6.2 Example: Hybrid cellular automaton of the bone ecosystem in cancer

Araujo and Basanta (2016) developed a hybrid cellular automaton for which the goal was to capture the ecosystem of the bone. A crude approximation of this ecosystem includes the bone itself, the myeloid‐derived cells such as osteoclasts that resorb bone, and the cells derived from mesenchymal stem cells, such as osteoblasts, that deposit new bone. Each of these cell types can be modelled as discrete agents regulated by diffusible factors—such as TGF‐β, RANK ligand, and other factors embedded in the bone matrix—described by partial differential equations. Parameterization of the model is facilitated by the fact that non‐cancerous cells have more predictable phenotypes, and the model's overall behaviour can be calibrated to ensure it recapitulates bone homeostasis. Araujo et al. (2018) thus studied how bone metastatic prostate cancer cells could infiltrate the bone ecosystem, take advantage of it, and grow. They also investigated what prostate cancer cells in the primary tumour should be of concern to physicians, and why conventional treatments that fail to disrupt tumour‐ecosystem interactions also fail to provide long‐term cancer cures in bone metastatic prostate cancer (Araujo et al., 2014).

### 6.3 Parameter inference

Unknown parameter values can be inferred by combining an SABM with a statistical method. This is, in fact, often the main objective of an SABM study. Approximate Bayesian computation is a popular approach that, in its simplest form, infers the value of a parameter θ as follows

1. From our data, calculate some summary statistic $\mu_{\text{data}}$;
2. Set i=1;
3. Run the model using a candidate parameter value $\theta_{i}$ drawn from some prior distribution;
4. Calculate the summary statistic $\mu_{i}$ for the model output;
5. If the difference between $\mu_{i}$ and $\mu_{\text{data}}$ is less than a predefined tolerance then add $\theta_{i}$ to the posterior distribution;
6. Increment i;
7. If i is less than some threshold then repeat from step 3.

Although simple in principle, approximate Bayesian computation requires careful implementation. The accuracy and precision of inferences depend on the choices of prior distributions, summary statistics and tolerances, as well as the number of iterations. Typically multiple parameter values cannot be precisely derived from prior data or models, in which case each should be assigned a vague (high variance) prior distribution. Tolerance values should be tuned such that neither too many nor too few candidate parameter values are accepted to the posterior distribution. Summary statistics should capture features of the system that provide useful information about the parameters of interest. A useful template is a 2010 study (Sottoriva & Tavaré, 2010) in which Sottoriva and Tavaré inferred aspects of stem cell dynamics in the colonic crypt by combining a cellular Potts model with approximate Bayesian computation, using a summary statistic based on methylation patterns.

An alternative to this approach was recently outlined in (Cess & Finley, 2023), in which the authors describe a novel method utilizing neural networks to reduce both tumour images and SABM simulations to low‐dimensional points. The distance between these points acts as a quantitative measure of how the two differ. This enables direct comparison, and by using parameter fitting algorithms to minimize the distance between the two sets of points, parameters can be estimated directly from the images and the simulations.

### 6.4 Sensitivity analysis

Whatever the objective, an essential step in any modelling study is to examine, as far as is practical, how the results and conclusions depend on uncertain aspects of the model. A common approach is to run a large number of model variants with different combinations of plausible parameter values. Varying one parameter at a time can provide useful insight into which parameters have the greatest impact on model output, with the shortcoming that non‐linear interactions between parameters are often neglected. A more sophisticated approach is to infer a multivariable “metamodel” function—a model of the model—that approximately describes how the model's parameters determine its outputs.

Since varying many parameters systematically on a continuous scale is infeasible, sampling methods such as Sobol sequencing (Sobol, 1967) or Latin hypercube sampling (McKay et al., 2000) can be used to generate a set of near‐randomly sampled combinations of parameter values. Both methods were used in a recent SABM study of cancer cell response to ATR‐inhibitors (Hamis, Yates, et al., 2021). A recent introductory review explains specifically how to apply these methods to cancer ABMs (Hamis, Stratiev, & Powathil, 2021). It is important to note that thorough sensitivity analysis involves varying not only parameter values but also mathematical relationships, aspects of spatial structure, and any other influential model components.

## 7 VISUALIZATION

Having built and parameterized a model, we next require useful ways to visualize its output. Typical methods represent spatial information, multidimensional phenotypic information, or evolutionary dynamics. Representing all these aspects in a single image is generally impossible.

### 7.1 Spatial plots

A spatial plot represents the state of an SABM at a moment in time. Producing a two‐dimensional grid plot of a two‐dimensional on‐lattice model is straightforward—we simply output the state of each site as a matrix of numbers and input this matrix into a bitmap (or raster) plotting function in R, Python, MATLAB, or similar software, using different colours to represent the different states (Figure 3). Our toy Eden growth model (Noble, 2018) provides an example implementation. Diffusible factor concentrations can be shown outside the tumour using a colour gradient and inside the tumour by adjusting brightness (Bacevic et al., 2017). We can apply the same method to off‐lattice models by specifying a grid and assigning each grid square a value that summarizes the states of all points within the square. Given multi‐level spatial structure, we can represent the most abundant state in each deme (Noble et al., 2022).

**FIGURE 5:**
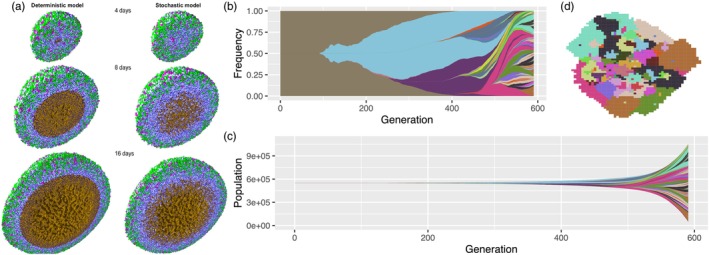
(a) Plots of a 3D off‐lattice ABM, produced in PhysiCell (Ghaffarizadeh et al., 2018), showing a cross‐section of model states of a hanging‐drop spheroid growth simulation at different time points, using either a deterministic or a stochastic SABM. Cells are coloured according to cell cycle position. Cells in the $K_{1}$ cell cycle state are green, post‐mitotic $K_{2}$ cells are magenta, quiescent cells are pale blue, apoptotic cells are red, and necrotic cells are brown. Cell nuclei are shown in dark blue. (b) Muller plot showing phylogenies and phenotype frequencies over time. (c) Fish plot showing phylogenies and phenotype population sizes over time. (d) 2D grid plot corresponding to same simulation as the Muller and fish plots in previous panels, with the same colour scheme, at the final time point. Plots b and c were produced using the R package *ggmuller* (Robert Noble, 2019). Image a is reproduced from (Ghaffarizadeh et al., 2018) under the terms of a Creative Commons Attribution License and with the approval of Paul Macklin. Plots (b–d) are reproduced from (Noble et al., 2020) under the terms of a Creative Commons Attribution License.

Illustrating three‐dimensional information is more technically demanding as we need to project the object onto a two‐dimensional plane, determine the visible surface, and add shading (as in Figure 5a). Suitable computational methods include rasterization and ray tracing, which can be performed in R and Python or using dedicated software, such as POV‐Ray. Further details can be found in the PhysiCell documentation (see Appendix S1). A much simpler solution is to plot only two‐dimensional slices.

### 7.2 Visualizing evolutionary dynamics

Muller plots represent subpopulation dynamics and phylogeny, while disregarding spatial information. The horizontal axis represents time and the vertical axis corresponds to subpopulation frequency. Each subpopulation is depicted as a shaded area emerging from its immediate ancestor (Figure 5b). Fish plots are similar but show population size rather than frequency (Figure 5c). Software packages for producing these plots include ggmuller (Robert Noble, 2019) and EvoFreq (Gatenbee et al., 2019).

### 7.3 Phenotype space plots

In a phenotype space plot, the axes correspond to continuous traits such as cell fitness, metabolic type and degree of drug resistance, and each point represents a cell. We can visualize phenotypic evolution by animating phenotype space plots from a series of time points. Robertson‐Tessi et al. (2015) pioneered the use of these plots in cancer research in their 2015 study of the effects of metabolic heterogeneity on tumour growth.

## 8 REPRODUCIBILITY

Reproducibility is a cornerstone of the scientific method. A reproducible modelling study not only allows others to easily regenerate its results but also permits further data processing, downstream analysis of generated data, generation of summary statistics, ease of production for visual representations or plots, and even adaptation of the existing model for novel purposes.

### 8.1 Principles of reproducible research

Gundersen (2021) describes three categories of reproducibility:

1. Outcome reproducibility: The reproduction experiment's result matches the original. If the same analysis of the result is performed, the same conclusions can be drawn, and the original hypothesis is supported by both experiments.
2. Analysis reproducibility: The reproduction experiment's result differs from the original, but if the same analysis method is used, the interpretation of the results still matches the original.
3. Interpretation reproducibility: The reproduction experiment's outcomes and the analysis of said outcomes both differ, but the interpretation matches the original interpretation.

Computational modelling studies should typically aim for the highest standard of outcome reproducibility. If care is taken to construct a well‐packaged computational study in a controlled digital environment, then in principle, given a suitable machine, the study should be easily reproduced exactly. This entails not only comprehensively explaining methods, results, analyses and interpretation, but also sharing the model code and scripts used at every step of pre‐processing and analysis, providing a detailed description of how to execute the code, and sharing any associated data and parameterization and configuration files.

In their outline of best practices to observe throughout a computational research project, Sandve et al. (2013) advocate tracking how every result is produced and reporting intermediate results as well as final outcomes. To make code easier to reproduce, one should catalogue the versions of software used at every point, record the seeds used in any random number generation, and implement version control (Heroux & Willenbring, 2009). Manual data manipulation should be avoided in favour of using automated methods to reformat and process raw data files. The raw data used to produce summary data plots should be easily accessible to facilitate easy plot reproduction and to allow readers to check individual data points. Textual descriptions of methods and results should link to the associated raw data and code so that a reader can easily follow all the steps leading to interpretations. Lastly, modellers are highly encouraged to share each full study, ideally with a dedicated public server. One such research‐oriented database is zenodo (Zenodo, 2023), where scientists may freely upload their research output permanently as a citeable piece of software.

### 8.2 Workflow managers, package managers and containers

A complex computational model will often require multiple steps to be carried out in sequence. If a high‐performance computing (HPC) cluster is required to run the model efficiently—as is typical for complex models—it is essential to utilize a workflow manager to properly orchestrate the steps (Wratten et al., 2021). Open‐source workflow managers allow researchers to package a model into a reproducible, cross‐platform workflow. Nextflow (Mölder et al., 2021) and Snakemake (Di Tommaso et al., 2017) are among the most popular workflow managers with several published pipelines (Cornwell et al., 2018; Hölzer & Marz, 2021; Kieser et al., 2020; Zhao et al., 2018), strong community support, and extensive documentation, giving users flexibility when designing their own custom pipelines. Snakemake is based on Python, a popular language among computational biologists and bioinformaticians. Nextflow uses the Java‐based language Groovy, which has a Python‐style structure and is relatively easy to for Python users to learn. Both also enable automatic parallelization for HPC clusters, which can be essential for complex SABMs or for running multiple instances of smaller models simultaneously.

Another option is to utilize container technologies, considered by many to be the gold standard in computational research. These are less computationally demanding than running an application on a computer directly or using a virtual machine and so permit faster deployment, patching and scaling. Containers also allow users to deploy the application on multiple operating systems or machines without reformatting and will run the application the same way no matter where they are deployed (Moreau et al., 2023). Docker (Merkel, 2014) is a popular container design platform which permits packaging applications into distribution‐independent containers. Another option, Bioconda (Grüning et al., 2018), enables easy dependency management, and can be deployed inside a container.

### 8.3 Extendable modelling platforms

For many research projects, the easiest option can be to build on an existing open‐source agent‐based modelling platform (see Appendix S1 for a brief guide). Some of these platforms—such as Chaste (Mirams et al., 2013), CompuCell3D (Swat et al., 2012), HAL (West, 2018) and PhysiCell (Ghaffarizadeh et al., 2018)—excel in simulating off‐lattice cell populations in complex microenvironments. Others, such as demon (Noble, 2019) (which has an automated computational workflow, Warlock (Bak et al., 2023)), J‐SPACE (Angaroni et al., 2022) and SMITH (Streck et al., 2023), focus on efficient modelling of evolutionary dynamics. Several are modular platforms, which facilitate reproducibility by making it easy to create and share extensions of the generic software. Nevertheless, even the most flexible platform is necessarily based on certain fundamental assumptions, structures and algorithms. If we want to create an especially innovative model, requiring several novel components that pre‐existing modelling platforms lack, then we might find it best to start from scratch. In principle, specialist rather than generalist models permit greater optimization in terms of memory demands and execution time.

### 8.4 FAIR principles in data management

As the volume of publicly available research data has been growing exponentially in recent decades (Statista, 2023), proper digital data management and annotation is recognized as an essential step in computational research—crucial for research reproducibility. Most notably, the FAIR principles have become a cornerstone in modern data management, particularly in the realms of scientific and research data (Wilkinson et al., 2016). FAIR is an acronym that encapsulates a set of guiding principles: Findable, Accessible, Interoperable, and Reusable. To be FAIR, data must first be Findable, meaning that it is easy for both humans and machines to discover, thanks to comprehensive metadata and proper indexing. Data should be Accessible, ensuring that access rights and permissions are clear and well‐defined, thus minimizing barriers to entry. Interoperable data is structured in a way that allows integration with other datasets by adhering to common standards, formats and vocabularies. Lastly, data should be Reusable, with thorough documentation, contextual information and availability in a format that facilitates easy replication and reuse. Altogether, the FAIR principles serve as a framework for enhancing data sharing, management and collaboration, ultimately driving scientific progress and fostering open science initiatives. Major organizations that have embraced FAIR guidelines include the European Open Science Cloud (EOSC, 2018), the European Life‐Science Infrastructure for Biological Information (ELIXIR, 2020), the US National Institutes of Health, and the Global Alliance for Genomics and Health (GA4GH, 2019).

## 9 DISCUSSION

Having surveyed the numerous choices that arise in any SABM project, we are faced with a problem: how can we choose the most appropriate model? In tumour evolution research, unlike in much of physics and engineering, there is no standard approach. Rather we must tailor a model to each research question by considering which components, events and interactions must be included, how far each aspect can be parameterized with available data, and the limits of our computational resources. It is essential to build on a sound understanding of the biological system and of the questions that matter to biologists and clinicians. Ideally this knowledge should come through close collaboration with empirical researchers throughout the model development process.

A general principle is that model complexity should match the complexity only of the phenomena of interest. We need not employ an off‐lattice hybrid SABM if a simple cellular automaton with only a few basic update rules can demonstrate the same principle. Attempting to represent every component of a biological system is not only computationally impractical but also risks overfitting and hinders explainability. Simpler models have many merits. They are easier to falsify and have fewer sources of potential error. They reduce researcher degrees of freedom and curb the tweaking of parameters to support a pet hypothesis. They are more mathematically tractable and easier to analyse. Perhaps most importantly, a simple model has wider applicability and can be more readily generalized, adapted or extended to answer new questions. More complicated models should be preferred only if the biological system is especially well understood or if simpler models have been tested and shown to be inadequate.

The greater difficulty—all too easily overlooked—is in choosing among a multitude of plausible simple models. An Eden growth model, for example, is arguably no more parsimonious than a spatial branching process or a spatial Moran process, which generate very different evolutionary dynamics. It is debatable whether the greater popularity of Eden growth models can be justified on biological grounds and is not simply due to them being easier to program.

Model design remains a challenge for even the most experienced researchers. One of the nine overarching themes in a recent review of key questions concerning the ecology and evolution of cancer (Dujon et al., 2021) was that we do not yet know which mathematical and computational models are the most useful. In another recent survey of cancer adaptive therapy modelling (West et al., 2023), four of the 11 key open questions were related to identifying appropriate mathematical models. When it comes to SABMs, the main limitations are twofold. First, we typically lack sufficient data to design and parameterize SABMs of large tumours. Second, routinely simulating much more than a few million individual cells (corresponding to no more than half a cubic centimetre of tumour) is computationally impractical. To some extent, these problems have technological solutions. Multi‐region sequencing, spatial multi‐omics, digital pathology and other modern methods are producing ever more detailed spatial tumour data. Accessible computing power continues to grow. But progress will also depend on developing smarter models.

Instead of drawing conclusions from a single SABM, we might do better to consider ensembles of models with diverse structures, algorithms and underlying assumptions. Much as in hurricane forecasting (Hamill et al., 2012), we can be more confident when many models converge on the same prediction. Another important direction is to develop coarse‐grained models that can simulate tumour evolution as accurately as cell‐level SABMs but with much greater computational efficiency. Rather than cell division, death, mutation and dispersal rates, coarse‐grained models depend on macroscopic parameters such as the arrival rate of consequential clones, clonal expansion speeds, and large‐scale microenvironmental heterogeneity. A potential way forward is to combine mathematical analysis of the relevant stochastic processes to determine appropriate approximations (Stein et al., 2024), and machine learning methods to infer the parameter values. SABMs capable of accurately simulating the evolution of entire tumours could have wide‐ranging applications, not least in patient‐specific clinical forecasting.

## CONFLICT OF INTEREST STATEMENT

The authors declare that there is no conflict of interest.

## Supporting information


Appendix S1

## Data Availability

Data sharing is not applicable to this article as no new data were created or analysed in this study.
