# Correction to “A seven‐step guide to spatial, agent‐based modelling of tumour evolution”

**DOI:** 10.1111/eva.13756

**Published:** 2024-07-09

Colyer, B., Bak, M., Basanta, D., & Noble, R. (2024). A seven‐step guide to spatial, agent‐based modelling of tumour evolution. *Evolutionary Applications*, 17, e13687.

The Acknowledgements section omitted to acknowledge funding from the National Cancer Institute of the National Institutes of Health under Award Number U54CA217376.

We apologize for this error.

