# Supplementary material for: A seven‐step guide to spatial, agent‐based modelling of tumour evolution
Source: Evol Appl. 2024 May 2;17(5):e13687. doi: 10.1111/eva.13687 (PMC11064804; doi:10.1111/eva.13687)
Supplement: Supplementary file 1 — Appendix S1 [file EVA-17-e13687-s001.pdf]

S1 APPENDIX OF A SEVEN-STEP GUIDE TO SPATIAL, AGENT-BASED MODELLING OF TUMOUR EVOLUTION

Spatial, agent-based modelling software

Cell-Based Chaste. The cell-based version of Chaste [1] is a highly sophisticated, multiscale computational framework for modelling cell populations. Chaste permits both on- and off-lattice models and has built-in code for simulation of specific biological systems, such as cancer development within colonic crypts. Chaste has its own ODE and PDE solver, called SUNDIALS [2].

CompuCell3D. CompuCell3D [3] is a general-purpose platform for implementing tissue development models, including the Glazier-Graner-Hogeweg (or cellular Potts) model that its developers pioneered. Its bespoke CC3D-Bionetsolver package solves ODEs and PDEs using a finite element method. CompuCell3D has been used in dozens of studies of cancer and morphogenesis.

Demon. Demon [4] specializes in simulating intratumour population genetics. Its multi-scale spatial structure makes it especially well suited to studying the evolution of glandular tumours. Demon can be configured to implement mathematically tractable models such as the Eden growth model, biassed voter model, spatial Moran process, and spatial branching processes. An automated computational workflow called warlock [5] facilitates running demon simulations in parallel on a high-performance computing cluster.

HAL. HAL [6] is a generic and highly customisable platform comprised of modular components which allow for multiple grids to operate simultaneously, each performing different tasks. For example, one grid might handle cell-cell interactions while another implements oxygen diffusion. HAL has multiple ODE and PDE solvers to suit different modelling needs. It also includes several pre-built model templates.

J-SPACE. J-SPACE [7] is a modelling platform designed specifically for phylogenetic modelling. It simulates cancer evolution on a grid (or some other graph) and generates synthetic reads from next-generation sequencing platforms. A primary goal of J-SPACE is to help researchers assess the impact of incomplete data or experimental error on downstream bioinformatics pipelines.

PhysiCell. PhysiCell [8] is a flexible framework that can implement physics-based off-lattice models of large numbers of cells in dynamic tissue microenvironments, with dynamic cell-cycle state tracking. PhysiCell uses a custom-built, open-source package for ODE and PDE solving, called BioFVM [9]. Potential functions are used to describe cell-cell interactions including adhesion, repulsion, and cell-matrix interactions [10].

SMITH. SMITH [11] implements a branching process with quasi-spatial constraints that separate the tumour into a proliferating shell and a static core. By simulating the dynamics of clones rather than individual cells, SMITH is able to simulate the evolution of a tumour up to a billion cells in only a few minutes on a standard desktop PC. This computational speed comes at the cost of the model's strong simplifying assumptions.

MORPHEUS. Morpheus [12] is a highly accessible open-source platform in which users can develop multi-scale, multicellular systems which couple ODEs, PDEs and cellular Potts models, with automatic scheduling. Rather than coding models manually, users can describe the model in biological and mathematical terms in Morpheus' GUI, and utilise provided tools for visualisation and parameter estimation.

References

[1] Mirams GR, Arthurs CJ, Bernabeu MO, Bordas R, Cooper J, Corrias A, et al. Chaste: an open source C++ library for computational physiology and biology. PLoS computational biology. 2013;9(3):e1002970.

[2] Hindmarsh AC, Brown PN, Grant KE, Lee SL, Serban R, Shumaker DE, et al. SUNDIALS: Suite of nonlinear and differential/algebraic equation solvers. ACM Transactions on Mathematical Software (TOMS). 2005;31(3):363–396.

[3] Swat MH, Thomas GL, Belmonte JM, Shirinifard A, Hmeljak D, Glazier JA. Multi-scale modeling of tissues using CompuCell3D. In: Methods in cell biology. vol. 110. Elsevier; 2012. p. 325–366.

[4] Noble R. demon; 2019. `https://github.com/robjohnnoble/demon_model`.

[5] Bak M, Colyer B, Manojlović V, Noble R. Warlock: an automated computational workflow for simulating spatially structured tumour evolution. arXiv preprint arXiv:230107808. 2023;.

[6] West J. Hybrid Automata Library;. `https://halloworld.org`.

[7] Angaroni F, Guidi A, Ascolani G, d'Onofrio A, Antoniotti M, Graudenzi A. J-SPACE: a Julia package for the simulation of spatial models of cancer evolution and of sequencing experiments. BMC bioinformatics. 2022;23(1):269.

[8] Ghaffarizadeh A, Heiland R, Friedman SH, Mumenthaler SM, Macklin P. PhysiCell: An open source physics-based cell simulator for 3-D multicellular systems. PLoS computational biology. 2018;14(2):e1005991.

[9] Ghaffarizadeh A, Friedman SH, Macklin P. BioFVM: an efficient, parallelized diffusive transport solver for 3-D biological simulations. Bioinformatics. 2016;32(8):1256–1258.

[10] Macklin P, Edgerton ME, Thompson AM, Cristini V. Patient-calibrated agent-based modelling of ductal carcinoma in situ (DCIS): from microscopic measurements to macroscopic predictions of clinical progression. Journal of theoretical biology. 2012;301:122–140.

[11] Streck A, Kaufmann TL, Schwarz RF. SMITH: spatially constrained stochastic model for simulation of intra-tumour heterogeneity. Bioinformatics. 2023;39(3):btad102.

[12] Starruß J, De Back W, Brusch L, Deutsch A. Morpheus: a user-friendly modeling environment for multiscale and multicellular systems biology. Bioinformatics. 2014;30(9):1331–1332.
